# Effect of Coiling Temperature on Microstructure, Properties and Resistance to Fish-Scaling of Hot Rolled Enamel Steel

**DOI:** 10.3390/ma10091012

**Published:** 2017-08-31

**Authors:** Yang Zhao, Xueqi Huang, Bo Yu, Xiaoyun Yuan, Xianghua Liu

**Affiliations:** 1 School of Materials Science and Engineering, Northeastern University, Shenyang 110819, China; 15832553619@163.com; 2 Shougang Research Institute of Technology, Beijing 100043, China; 3 State Key Laboratory of Rolling and Automation, Northeastern University, Shenyang 110819, China; caijiayubo@sina.com (B.Y.); liuxh@mail.neu.edu.cn (X.L.); 4 College of Mechanical and Electrical Engineering, Xi’an Polytechnic University, Xi’an 710048, China; xiaoyun_yuan@126.com

**Keywords:** hot rolled enamel steel, coiling temperature, microstructure, resistance to fish-scaling, interphase precipitates

## Abstract

The microstructure, mechanical properties, and hydrogen permeation behavior of hot rolled enamel steel were investigated. Three coiling temperatures were adopted to gain different sizes of ferrite grain and TiC precipitates. The results show that a large number of interphase precipitates of nano-sized TiC can be obtained at coiling temperatures of 650 and 700 °C, while a few precipitates are found in experimental steel when coiling temperature is 600 °C. The yield strength and ultimate tensile strength decrease with increasing coiling temperature, while elongation increases. The experimental steel has the best resistance to fish-scaling at coiling temperature of 700 °C, due to the large quantities of nano-sized interphase precipitates of TiC.

## 1. Introduction

Porcelain enamel coatings for steels possess chemical and mechanical stability in various environments, including acid, alkaline, high-temperature and harsh working conditions, and so are widely used for a variety of consumer applications and for the protection of steel in many industrial chemical applications [1]. Hot rolled enamel steels are widely applied to water treatment equipment, chemical reactor, and environmental protection equipment due to their excellent corrosion resistance and sufficient yield strength for engineering design. 

Fish-scaling is one of the most dangerous and common defects in the production of enameled steel products. Fish-scaling has been defined as the development of a considerable amount of hollow semicircles on the surface of coated items that together resemble fish scales, and which exert a detrimental surface aspect [2]. It is caused by an excess of hydrogen which dissolves into the steel during the enameling process, especially during the enamel firing at the temperature of 800–850 °C [3]. Since its solubility dramatically decreases during the following cooling process, hydrogen moves toward the steel-enamel interface in quantities that cause fish-scaling even after a lapse of time. 

Sufficient hydrogen traps are needed to ensure good resistance to the fish-scaling of steels. Precipitates are the main irreversible hydrogen traps in hot rolled enamel steels. Large quantities of precipitates mean good resistance to fish-scaling. Challa et al., revealed that the distribution and the number of precipitates was a function of coiling temperature [4]. That is to say, excellent resistance to fish-scaling of hot rolled enamel steels can be obtained through controlling coiling temperature. In this work, the effect of coiling temperature on microstructure and properties of hot rolled enamel steel was investigated. The purpose of this work is to elucidate the relationship between resistance to fish-scaling and microstructure of hot rolled enamel steel.

## 2. Materials and Methods

The chemical composition of experimental steel is listed in Table 1. The experimental steel was melted by a vacuum induction furnace and cast to a 150 kg ingot. The ingot was forged to billets with a section of 50 mm × 120 mm. They were reheated to 1200 °C and held for 2 h, then hot rolled to sheets of 5 mm thick at finishing temperature of 900 °C. The sheets were subsequently water cooled to coiling temperatures, and the coiling temperatures were 600, 650 and 700 °C, respectively.

Metallographic specimens were cut along the transverse direction, then were polished and etched with a 4% nital solution before the observation by means of Leica DMIRM 2500 optical microscope (OM, Leica, Wetzlar, Hesse, Germany). Transmission electron microscope (TEM) observations were carried out on 3 mm diameter thin foils by FEI Tecnai G^2^ F20 TEM (FEI, Houston, TX, USA) at an acceleration voltage of 200 kV. Thin foils were prepared by twin-jet electropolishing using 10% perchloric acid in ethanol. 

Tensile specimens with size of 50 mm × 12.5 mm were machined from the sheet along the transverse direction. Standard tensile tests were conducted at room temperature by using a crosshead speed of 3 mm/min on a CMT5105 tensile testing machine (MTS, Shenzhen, Guandong, China).

A hydrogen permeation test was conducted at 25 °C by using electrochemical method developed by Devanathan and Stachurski [5]. Square specimens with area of 50 mm × 50 mm were cut from the hot rolled steel sheets. The specimens were degreased with acetone and then cleaned in distilled water. The hydrogen permeation setup was composed of two parts separated by the specimen into the hydrogen charging cell and hydrogen releasing cell. The cells were purged with high-purity nitrogen gas to remove the dissolved oxygen from the solutions before the hydrogen permeation test [6]. The specimen was fixed on the electrochemical cell using an O-type ring. The charging cell was filled with 500 mL 0.5 M H_2_SO_4_ + 0.22 g/L H_2_NCSNH_2_ solution, and the releasing cell was filled with 500 mL 0.2 M NaOH solution. In the charging cell, hydrogen atoms were introduced by cathodic charging (H^+^ + e→H). H_2_NCSNH_2_ facilitated hydrogen pick-up by promoting the breakdown of molecular hydrogen. The hydrogen oxidation (H→H^+^ + e) current can be measured in the releasing cell under an applied constant anodic potential. 

**Figure 1:**
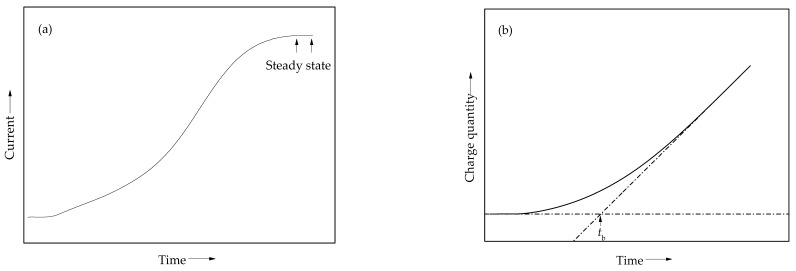
Typical hydrogen permeation curves (**a**) *I*-*t* curve; (**b**) *Q*-*t* curve.

The determination method of hydrogen permeation time is described in reference [7]. The typical hydrogen permeation curves are shown in Figure 1. The current-time curve (*I*-*t* curve) of hydrogen permeation is shown in Figure 1a. The current increases slowly at the initial stage of the test, and then increases quickly, finally achieves a steady state. The charge quantity-time curve (*Q*-*t* curve) can be obtained by integrating the *I*-*t* curve, as shown in Figure 1b. Through the lowest point of the *Q*-*t* curve, making a line parallel to the *X*-axis. The steady state section of the *I*-*t* curve corresponds to the linear section of the *Q*-*t* curve, making a tangent line to the linear section. The two lines intersect each other, and the abscissa value of the intersection point is the hydrogen permeation time. The hydrogen permeation value (*TH*) can be calculated by using the following formula.

(1) $$TH=\frac{t_{b}}{d^{2}}$$

where *t_b_* is the hydrogen permeation time in minutes; *d* is the sheet thickness in mm. Large hydrogen permeation value means high hydrogen storage capacity and good resistance to fish-scaling of enamel steel. 

Ma et al. revealed that the *TH* value was a constant when the thickness of sheet was larger than 0.6 mm [8]. The specimens for the hydrogen permeation test were machined to 2.8 mm in thickness in order to reduce the test duration. 

## 3. Results and Discussion

### 3.1. Microstructure

**Figure 2:**
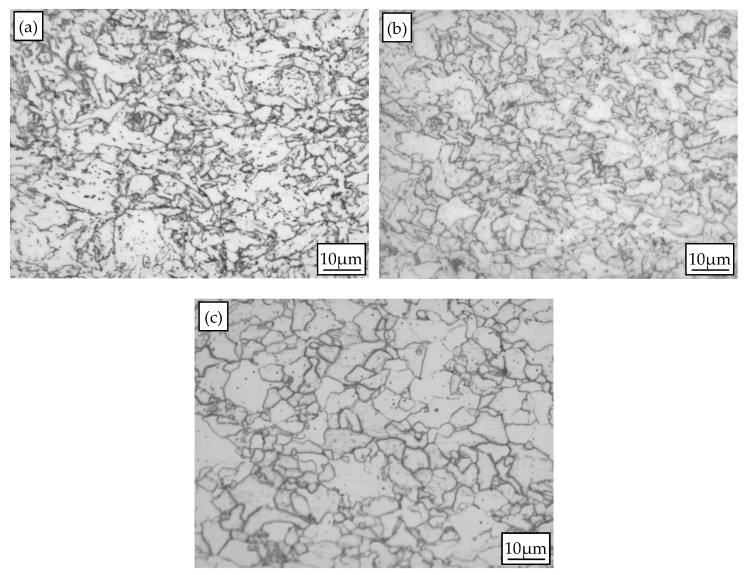
OM micrographs of experimental steel coiled at different temperatures: (**a**) 600 °C; (**b**) 650 °C; (**c**) 700 °C.

Figure 2 shows OM micrographs of experimental steel coiled at different temperatures. All the microstructures were composed of ferrite and fine carbides. The ferrite grains are elongated when coiling temperatures are 600 and 650 °C, and the average ferrite grain sizes are smaller than those of experimental steel coiled at 700 °C. The polygonal ferrite grain indicates that recrystallization has occurred when coiling temperature is 700 °C. 

**Figure 3:**
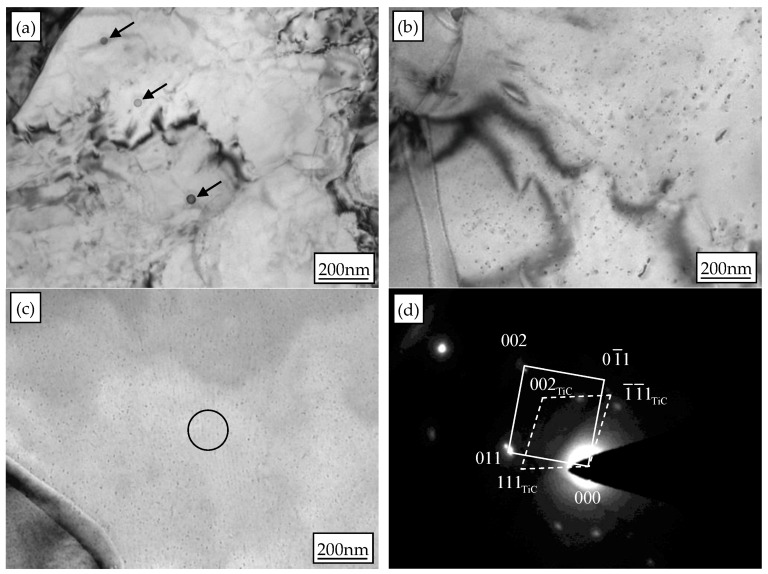
TEM images and SADP of precipitates of experimental steel: (**a**) coiled at 600 °C; (**b**) coiled at 650 °C; (**c**) coiled at 700 °C; (**d**) SADP of interphase precipitates in (**c**).

The morphology and the distribution of the precipitates in experimental steel are shown in Figure 3. There are a few randomly dispersed precipitates when coiling temperature is 600 °C, and the average diameter of precipitates is about 35 nm. This kind of precipitate is believed to form during solidification, rather than during the coiling process. When coiling temperatures are 650 and 700 °C, there are a large number of interphase precipitates, which indicates that the coiling temperature significantly affects the precipitation behavior of precipitates containing Ti. Interphase precipitation is associated with discrete particles distributed in planar sheets, which lie approximately parallel to the advancing *γ*→*α* transformation front. The selected area diffraction pattern (SADP) analysis of interphase precipitates in Figure 3c reveals that the precipitated particles are TiC carbides. The TiC particles have a NaCl-type crystal structure and conform to the Baker-Nutting (B-N) orientation relationship with respect to the ferrite matrix [9]; (001)_TiC_//(001)*_α_* and [110]_TiC_//[001]*_α_*. This also indicates that the interphase precipitates are associated with a semi-coherent relationship at (002)_TiC_//(002)*_α_* interface with the *α* matrix. The average diameter of interphase precipitates at a coiling temperature of 650 °C is about 8 nm, while the average diameter of interphase precipitates at a coiling temperature of 700 °C is about 5 nm.

Kim et al. also found that the coiling temperature played an important role in the precipitation behavior of hot rolled steels [10]. Xu et al. implied that interphase precipitation occurred at a coiling temperature of 640 °C, while randomly dispersed precipitation formed at 600 °C [11]. These results are consistent with our observations. At a coiling temperature of 600 °C, the growth of ferrite is too rapid, not allowing enough time for interphase precipitation to take place [12]. For the rolling schedule of experimental steel, *γ*→*α* transformation can take place during the rolling process; thus, interphase precipitation is preferred as a precipitation mechanism, rather than strained induced precipitation [10]. Large quantities of precipitates can ensure good thermal stability of experimental steel during the enamel firing process, due to the low solubility of precipitates containing Ti at firing temperature of 800–850 °C. 

### 3.2. Mechanical Properties

The mechanical properties of experimental steel coiled at different temperatures are listed in Table 2. It can be seen that both yield strength (YS) and ultimate tensile strength (UTS) decrease with increasing coiling temperature, while elongation increases. As high coiling temperature can lead to large ferrite grain and small size of precipitates, it can be concluded that the contribution of ferrite grain size to strength is larger than that of precipitates. 

### 3.3. Resistance to Fish-Scaling

**Figure 4:**
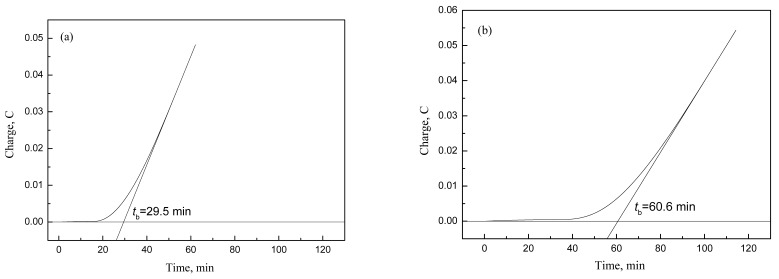

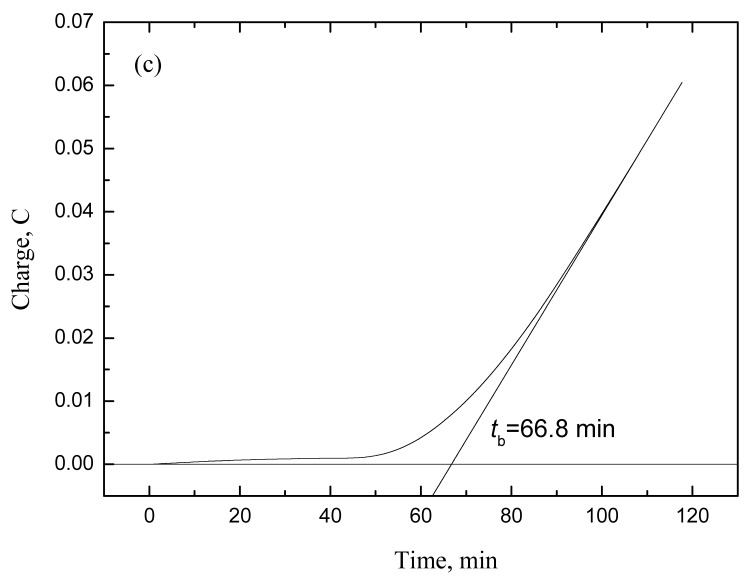
Hydrogen permeation curves of hot rolled enamel steel coiled at different temperatures: (**a**) 600 °C; (**b**) 650 °C; (**c**) 700 °C.

The hydrogen permeation curves (*Q*-*t* curves) of experimental steel are shown in Figure 4. The hydrogen permeation times of specimens coiled at 600, 650 and 700 °C are determined to be 29.5, 60.6 and 66.8 min, respectively. The corresponding thicknesses of specimens are 2.8, 2.81 and 2.82 mm, respectively, so the *TH* values of hot rolled enamel steel coiled at 600, 650 and 700 °C are calculated to be 3.8, 7.7 and 8.4 min/mm^2^, respectively. The resistance to fish-scaling of experimental steel is found to increase as coiling temperature increases. It has been reported that steels have satisfactory resistance to fish-scaling when *TH* value is larger than 6.7 min/mm^2^ [13]. Obviously, the experimental steel has satisfactory resistance to fish-scaling when coiling temperatures are 650 and 700 °C. The experimental steel has the best resistance to fish-scaling when coiling temperature is 700 °C. 

There are two kinds of hydrogen traps—reversible and irreversible traps—depending on their binding energies with hydrogen atoms. A site with high binding energy is considered to be an irreversible trap. A reversible trap is one from which a hydrogen atom can easily jump out, due to fluctuations in thermal energy [14]. Vacancies, dislocations, and grain boundaries are reversible traps due to their low binding energies. Coherent precipitates, such as (Ti, Nb) (C, N), TiC, TiN, NbC and VC, are considered to be irreversible hydrogen traps [15]. Grain boundaries and precipitates are the main hydrogen traps in experimental steel. As mentioned above, experimental steel has more grain boundary areas and less precipitates when the coiling temperature is 600 °C, while the opposite is true when the coiling temperature is 700 °C. Considering that the *TH* value is highest when the coiling temperature is 700 °C, it can be concluded that the resistance to fish-scaling is influenced significantly by precipitates. Compared to the large size of randomly dispersed precipitates, nano-sized interphase precipitates can effectively enhance the resistance to fish-scaling.

The trapping characteristics of precipitates depend on their size, morphology, quantities and nature of the matrix-precipitates interface. Wei et al. [16] insisted that the (semi-)coherent and incoherent TiC precipitates have distinctly different hydrogen trapping behaviors. The main hydrogen trapping sites of incoherent precipitates are the disordered interfaces and carbon vacancies inside the precipitates. Incoherent TiC particles absorb hydrogen at high temperatures, but they are not able to trap hydrogen at room temperature during cathodic charging due to the high energy barrier for trapping. In contrast, (semi-)coherent TiC particles can trap hydrogen during cathodic charging at room temperature. The cores of misfit dislocations at the {100}_TiC_//{100}_Fe_ interfaces are the main trapping sites for the semi-coherent precipitates [16,17]. Moreover, the amount of hydrogen trapped by (semi-)coherent TiC particles depends on the amount of interfacial area. A larger interfacial area means more trapped hydrogen and higher resistance to fish-scaling. Takahashi et al. observed the hydrogen trapping sites of nano-sized TiC precipitates by using atom probe tomography for the first time [18]. They found that the main trapping sites of coherent TiC precipitates were the matrix-precipitate interfaces. In our study, the TiC particles coiled at 700 °C have a smaller average diameter and, resultantly, a larger interfacial area than those of TiC coiled at 650 °C. Considering that the *TH* value of experimental steel coiled at 700 °C is larger than that of experimental steel coiled at 650 °C, it can be concluded that the *TH* value is related to the area of the interface between TiC particles and the ferrite matrix. This result is consistent with the results reported by Takahashi et al. [18] and Wei et al. [16]. Because the precipitation behavior of TiC can be controlled by varying the coiling temperature, the resistance to fish-scaling of hot rolled enamel steel can be improved by optimizing the coiling temperature. 

## 4. Conclusions

In this study, three coiling temperatures were employed to investigate its effect on the microstructure, mechanical properties and resistance to fish-scaling of hot rolled enamel steel. TEM observation was conducted to clarify the precipitation behavior under different coiling temperatures. A hydrogen permeation test was employed to evaluate the resistance to fish-scaling. The following conclusions can be drawn.

(1) At coiling temperatures of 650 and 700 °C, there are large quantities of interphase precipitates of TiC in hot rolled enamel steel. While at coiling temperature of 600 °C, there are a few randomly dispersed precipitates containing Ti.
(2) Coiling at low temperature, the microstructure was characterized by elongated ferrite grain. On the other hand, coiling at high temperature can lead to polygonal ferrite and low yield strength and ultimate tensile strength.
(3) At a temperature range of 600–700 °C, the resistance to fish-scaling is best when the coiling temperature is 700 °C, because of the large quantities of nano-sized interphase precipitates of TiC in hot rolled enamel steel. The main irreversible hydrogen trapping sites are matrix-TiC interfaces, and nano-sized interphase precipitates are beneficial to the improvement of the resistance to fish-scaling.

## Figures and Tables

**Table 1:** Chemical composition of experimental steel (wt %).

C	Si	Mn	P	S	Al	Ti	Fe
0.07	0.028	1.2	0.004	0.005	0.012	0.13	Bal.

**Table 2:** Mechanical properties of experimental steel coiled at different temperatures.

Coiling Temperature (°C)	YS (MPa)	UTS (MPa)	Elongation (%)
700	493	598	32.8
650	535	605	20.2
600	560	611	19.4
